# Role of B7-H4 siRNA in Proliferation, Migration, and Invasion of LOVO Colorectal Carcinoma Cell Line

**DOI:** 10.1155/2015/326981

**Published:** 2015-05-21

**Authors:** Hai-xia Peng, Wei-qi Wu, Da-ming Yang, Rong Jing, Ji Li, Feng-li Zhou, Yun-fei Jin, Sai-yu Wang, Yi-min Chu

**Affiliations:** Digestive Endoscopy Center, Shanghai Tongren Hospital, Shanghai Jiao Tong University School of Medicine, 1111 Xianxia Road, Shanghai 200336, China

## Abstract

*Objectives*. Colorectal cancer is one of the most common malignancies. Recent studies investigated that B7-H4 is highly expressed in various cancers. We aimed at exploring the effect of B7-H4 siRNA on proliferation, invasion, and migration of LOVO cells which expressed B7-H4 notably.* Design and Methods*. Colon adenocarcinoma dataset was downloaded from The Cancer Genome Atlas. 35 colorectal cancer patients admitted to Shanghai Tongren Hospital were enrolled in this study. Cell proliferation and cell cycle distribution were identified by CCK8 and flow cytometry, respectively. Transwell assay was performed to detect the invasion and migration of LOVO cells. CXCL12/CXCR4 expression and JAK2/STAT3 phosphorylation were determined by real-time PCR and western blot.* Results*. B7-H4 expressed is elevated in colorectal cancer tissues than in the adjacent normal tissues. B7-H4 siRNA effectively inhibited the proliferation at 24 h and 48 h, arrested cell cycle at G0/G1, and suppressed cell invasion and migration. Gene set enrichment analysis showed that CXCL12/CXCR4 and JAK/STAT were correlative with the B7-H4 expression. Additionally, CXCL12/CXCR4 expression and JAK2/STAT3 phosphorylation were reduced.* Conclusions*. B7-H4 siRNA can effectively inhibit proliferation, invasion, and migration of LOVO cells by targeting CXCL12/CXCR4 and JAK2/STAT3 signaling, which can serve as a new target for colorectal carcinoma treatment.

## 1. Introduction

Colorectal cancer is one of the most common malignancies encountered in the world and is the second most common cause of cancer-related mortality [1, 2]. In the past several decades, the incidence of colorectal carcinoma has also risen in the whole world. With the development of diagnostic technology in recent years, increasing number of patients are diagnosed with early-stage colon cancer, and proper treatment was taken in time. However, there are still patients with early surgery suffering from metastases especially colorectal liver metastases, which can finally result in death [3]. Therefore, focusing on the molecular mechanism of colorectal liver metastases is the pressing target in colorectal carcinoma therapy.

B7 family molecules, including B7-H1 (PD-L1), B7-DC (PD-L2), and B7-H4 (B7S1), combine with the corresponding receptors of activated T lymphocytes, which negatively regulate T cell immunity [4]. This plays an important role in downregulating antitumor immunity in cancer microenvironments. B7-H4 is a newly discovered transmembrane protein of the B7-CD28 family. It is expressed in human tissues in the cancers of the colon, lung, pancreas, brain, stomach, breast, ovary, esophagus, prostate, liver, and kidney [5–7], which is classified as coinhibitors of cell-mediated immunity [5]. It combines with surface receptors of the activated T cell, thus negatively regulating T cells' growth, cell cycle, and immune function. Recently, B7-H4 was found to be highly expressed in colorectal carcinoma [8]. As a consequence, we attempt to explore the effect of the B7-H4 siRNA silencing on proliferation, cell circle distribution, migration, and invasion in colorectal cancer LOVO cells. To gain further insight into the biological pathways involved in colorectal cancer pathogenesis through B7-H4 pathway, a gene set enrichment analysis (GSEA) was performed.

Presently, we demonstrated the effect of B7-H4 siRNA on proliferation, cell circle distribution, invasion, and migration. GSEA showed that CXCL12/CXCR4 and JAK/STAT were correlative with the B7-H4 expression. The expression of CXCL12, CXCR4, p-JAK2, JAK2, p-STAT3, and STAT3 was also studied by means of RT-PCR and western blot analysis.

## 2. Materials and Methods

### 2.1. Bioinformatics Analysis

The gene expression data were obtained at The Cancer Genome Atlas website (TCGA, https://tcga-data.nci.nih.gov/tcga/) for the colon adenocarcinoma (COAD) projects. To gain further insight into the biological pathways involved in colorectal cancer pathogenesis through B7-H4 pathway, a gene set enrichment analysis (GSEA) was performed. GSEA is a method of analyzing and interpreting microarray and such data using biological knowledge [9]. The data in question is analyzed in terms of their differential enrichment in a predefined biological set of genes (representing pathways). These predefined biological sets can be published information about biochemical pathway or coexpression in a previous experiment. GSEA was performed using GSEA version 2.0 from the Broad Institute at MIT. The TCGA data were analyzed by GSEA. In this study, GSEA firstly generated an ordered list of all genes according to their correlation with B7-H4 expression and then a predefined gene set (signature of gene expression upon perturbation of certain cancer-related gene) receives an enrichment score (ES), which is a measure of statistical evidence rejecting the null hypothesis that its members are randomly distributed in the ordered list. The expression level of B7-H4 was used as phenotype label, and “metric for ranking genes” was set to Pearson's correlation. All other basic and advanced fields were set to default. The KEGG gene sets, biological process database (c2.KEGG.v4.0) from the Molecular Signatures Database–Msig DB (http://www.broad.mit.edu/gsea/msigdb/index.jsp), were used for enrichment analysis.

### 2.2. Patients and Tissue Samples

35 colorectal cancer patients admitted to Shanghai Tongren Hospital were enrolled in this study. All patients have complete clinical and pathological follow-up data. Adjacent normal colorectal tissues were also collected as negative controls. These normal colorectal tissues were resected within at least 5 cm of the tumor margin when the patients underwent definitive surgery. Ethical approval for the study was provided by the independent ethics committee, Shanghai Tongren Hospital. Informed and written consent was obtained from all patients or their advisers according to the ethics committee guidelines.

### 2.3. Cell Culture

LOVO, SW480, HCT116, SW620, and HT29 cells are human colorectal carcinoma cells. All cells were obtained from the Shanghai Cell Bank, Chinese Academy of Sciences (Shanghai, China). Cells were cultured in DMEM supplemented with 10% fetal bovine serum, 100 units/mL penicillin, and 100 *μ*g/mL streptomycin and incubated in a humidified atmosphere at 37°C with 5% CO_2_.

### 2.4. miRNA Transfection

LOVO cells were seeded in antibiotic-free medium the day before transfection. The cells were transfected with 50 nmol/L of B7-H4 siRNA or negative control by using lipofectamine 2000 (Invitrogen, Shanghai, China) according to the instructions provided by the manufacturer. After 48 hours, the transfected cells were collected and processed for quantitative real-time PCR (qPCR), western blot, proliferation, cell cycle, migration, and invasion assay.

### 2.5. Cell Proliferation Assay

Cell viability was assessed by Cell Counting Kit- (CCK-) 8 (Tongren, Shanghai, China). Briefly, 4 × 10^3^ cells were seeded in each 96-well plate and further incubated for 24 and 48 hours, respectively. CCK-8 reagent was added to each well at 1 hour before the endpoint of incubation. The optical density (OD) 450 nm values in each well were determined by a microplate reader. Experiments were repeated at least three times each time in triplicate.

### 2.6. Cell Cycle Assay

After 48 hours of transfection, cells were harvested and cell cycle distribution was analyzed using flow cytometer (FACSCalibur, BD Biosciences, Franklin Lakes, NJ, USA). DNA histograms were analyzed using flow cytometer (FACSCalibur, BD Biosciences).

### 2.7. Cell Invasion Assay

Cell invasion assay was performed by a 24-well Transwell chamber with a pore size of 8 *μ*m (Sigma, San Francisco, USA). The inserts were coated with 50 *μ*L Matrigel (dilution at 1 : 2; BD Bioscience). Cells were trypsinized after transfection for 24 hours and transferred to the upper Matrigel chamber in 100 *μ*L of serum-free medium supplementing 1 × 10^5^ cells and incubated for 24 hours. The lower chamber was filled with medium containing 10% FBS as chemoattractants. After incubation, the cells that passed through the filter were fixed and stained by 0.1% crystal violet. The numbers of invaded cells were counted in five randomly selected high power fields under a microscope (OLYMPUS, Shenzhen, China).

### 2.8. Migration Assay

Cells in logarithmic phase were digested by 0.25% trypsin (Gibco, Shanghai, China) and then suspended in RPMI-1640 (HyClone) medium containing 10% fetal calf serum (Gibco). Cells were seeded in a 12-plate microplate at a density of 1 × 10^5^ cells/mL and then incubated for 1 h. The supernatant was discarded and cells were washed 2 times by PBS (Gibco). 4% paraformaldehyde (JRDUN Biotech, Shanghai, China) was supplemented for 15 min and cells were stained by Giemsa (JRDUN) for 30 min. Then, cells were washed several times and the optical density (OD) values were read at 570 nm by a microplate reader (Thermo, Waltham, USA). Adhesion rate (%) = (OD_1_/OD_0_) × 100%, with OD_1_ being HB treated groups and OD_0_ being control group.

### 2.9. Reverse Transcription and Real-Time PCR

The mRNA expression of B7-H4 in LOVO cells after B7-H4 siRNA transfection was quantified by RT-PCR. Total RNA was isolated from cells using Trizol Reagent (Invitrogen) and quantified. cDNA was synthesized from 5 mg of RNA using AMV reverse transcriptase (Fermentas, USA) according to the manufacturer's instructions. B7-H4 was amplified from the cDNA by RT-PCR. The PCR conditions consisted of 5 min at 95°C one cycle, 30 seconds at 95°C, 30 seconds at 55°C, 30 seconds at 72°C, and 7 min at 72°C 35 cycles. Primer pairs for human genes were designed using the Primer Express Software (Applied Biosystems, Shanghai, China) and are listed in Table 1.

### 2.10. Western Blot

Cultured or transfected cells were harvested and washed twice with PBS and lysed in ice-cold radio immunoprecipitation assay buffer (RIPA, Beyotime, Shanghai, China) with freshly added 0.01% protease inhibitor cocktail (Sigma, Shanghai, China) and incubated on ice for 30 min. Cell lysis was centrifuged at 13,000 rcf for 10 min at 4°C and the supernatant (20–30 *μ*g of protein) was run on 10% SDS-PAGE gel and transferred electrophoretically to a polyvinylidene fluoride membrane (Millipore, Shanghai, China). The blots were blocked with 5% skim milk, followed by incubation with antibodies against B7-H4 (Abcam), GAPDH (Fermentas), CXCL12 (Abcam), CXCR4 (Abcam), p-JAK2 and JAK2 (CST), and p-STAT3 and STAT3 (CST). Blots were then incubated with goat anti-mouse or anti-rabbit secondary antibody (Beyotime, Shanghai, China) and visualized using enhanced chemiluminescence (ECL, Thermo Scientific, Shanghai, China).

### 2.11. Growth of Cells in Athymic Nude Mouse and Tumor Size Determination

LOVO cells transfected with B7-H4 siRNA and cells with nontreatment were trypsinized and were washed and resuspended in DMEM without FBS. Cell concentration and viability were determined using trypan blue. Eight male athymic nude mice were randomly divided into 2 groups (4 mice/group) and were subcutaneously injected by 2 × 10^6^ cells transfected with control cells or B7-H4 siRNA cells, respectively. The tumor size was determined every 4 days after tumor formed (around 1-2 weeks) as previously described [10]. 48 days later, the mice were sacrificed and photographed, and the tumors were weighted on a digital balance.

### 2.12. Statistical Analysis

All results are presented as the mean ± SD and the data were analyzed by a SPSS 13.0 statistical package (SPSS Inc., Chicago, IL). Data for multiple comparisons were subjected to one-way ANOVA followed by Dunnett's test and a value of *p* < 0.05 was considered statistically significant.

## 3. Results

### 3.1. High Expression of B7-H4 in Tumor Tissue

**Figure 1:**
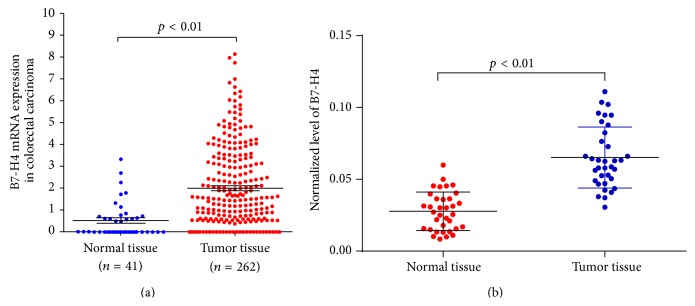
B7-H4 expression in colon cancer tissues and their adjacent normal tissues. 35 pairs of colon cancer tissues and their adjacent normal tissues were collected and mRNA expression of B7-H4 mRNA expression was identified by RT-PCR.

The gene expression data were obtained at The Cancer Genome Atlas website (TCGA, https://tcga-data.nci.nih.gov/tcga/) for the colon adenocarcinoma (COAD) projects. As shown in Figure 1(a), a notable increase was observed in B7-H4 mRNA expression of colorectal carcinoma tissue compared with normal tissue.

To further verify the biological role of B7-H4 in colorectal carcinoma, we used real-time PCR to detect the expression levels of B7-H4 in colorectal cancer patients' tissues. Because the expression of B7-H4 is considered quite low in gastrointestinal cancer, B7-H4 levels in ovarian cancer and normal tissues (*n* = 10) were also detected as positive control (see Figure S1 in Supplementary Material available online at http://dx.doi.org/10.1155/2015/326981). As Figure 1(b) shows, B7-H4 expression level was higher in colorectal tumor tissues than that in adjacent normal tissue control (*n* = 35, *p* < 0.01).

### 3.2. B7-H4 Expression in Colorectal Carcinoma Cell Lines

**Figure 2:**
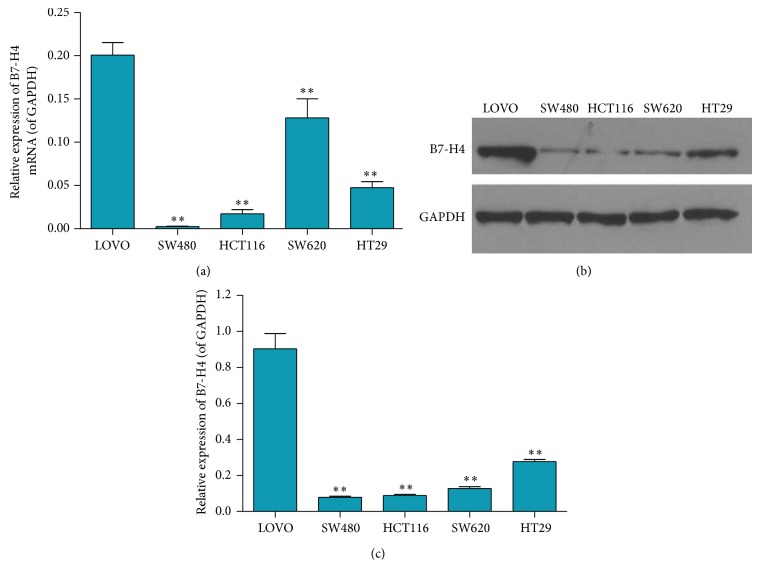
B7-H4 expression in colorectal carcinoma LOVO, SW480, HCT116, SW620, and HT29 cell lines. (a) B7-H4 mRNA expression was determined by RT-PCR in LOVO, SW480, HCT116, SW620, and HT29 cell lines. (b and c) Protein levels of B7-H4 were determined by western blot in LOVO, SW480, HCT116, SW620, and HT29 cell lines. ^*∗∗*^ *p* < 0.01 compared with the LOVO cells; data are expressed as the mean ± SD, *n* = 6.

An obvious difference was presented in colorectal cancer tissue and normal tissue. We then detected the mRNA expression and protein level of B7-H4 in various colorectal carcinoma cell lines including LOVO, SW480, HTC116, SW620, and HT29 by RT-PCR and western blot, respectively. As shown in Figure 2(a), B7-H4 mRNA expression in LOVO cell line was significantly higher than any other cell line. In addition, western blot displayed that protein level of B7-H4 was the highest among all cell lines (Figures 2(b) and 2(c)). As a result, LOVO cell line was determined to carry out further investigations.

### 3.3. Effect of B7-H4 siRNA on Cell Viability of LOVO Cell Line

**Figure 3:**
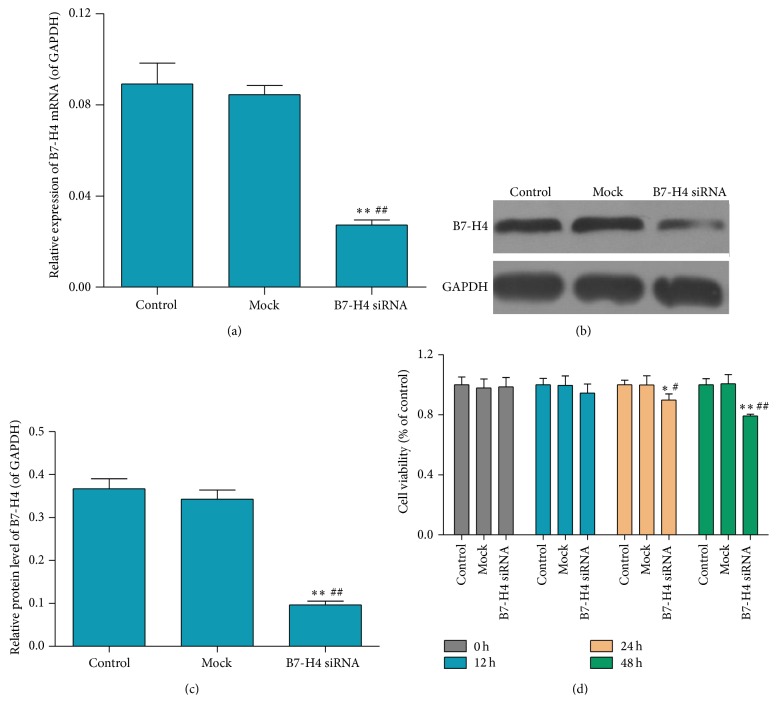
Effect of B7-H4 siRNA on cell viability of LOVO cells. (a) mRNA expression of B7-H4 in LOVO cells after B7-H4 siRNA transfection for 48 h was quantified by RT-PCR. (b and c) Protein expression of B7-H4 in LOVO cells after B7-H4 siRNA transfection for 48 h was quantified by western blot analysis. (d) After B7-H4 siRNA transfection for 0, 12, 24, and 48 h, cell viability of LOVO cells was identified by flow cytometry. ^*∗∗*^ *p* < 0.01 compared with the control cells; ^##^ *p* < 0.01 compared with the mock cells; data are expressed as the mean ± SD, *n* = 6.

B7-H4 mRNA was interfered in LOVO cell line as previously described. RT-PCR and western blot were employed to identify the interference efficient.  Figure 3(a) showed that B7-H4 mRNA expression in B7-H4 siRNA group was decreased dramatically compared with the control group and mock group. Western blot showed that protein level was declined notably in B7-H4 siRNA group in comparison with the control group and mock group (Figures 3(b) and 3(c)).

B7-H4 siRNA on cell viability of LOVO cell line was measured by CCK-8 assay. In Figure 3(d), cell viability was weakened in B7-H4 siRNA group markedly after transfection, 24 h and 48 h, in comparison with that of control and mock cells.

### 3.4. Effect of B7-H4 siRNA on Cell Cycle of LOVO Cell Line

**Figure 4:**
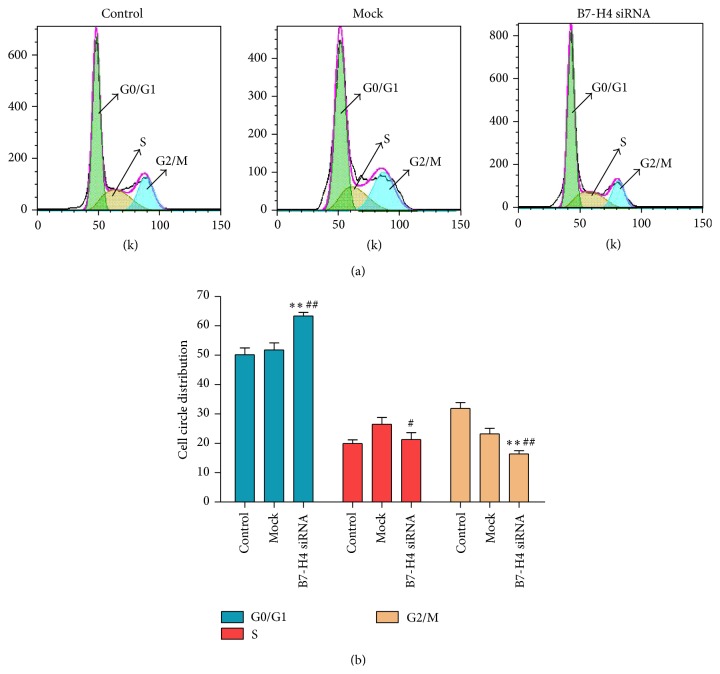
Effect of B7-H4 siRNA on cell circle distribution of LOVO cells. (a and b) After B7-H4 siRNA transfection for 48 h, cell circle distribution of LOVO cells was identified by flow cytometry. ^*∗∗*^ *p* < 0.01 compared with the control cells; ^#^ *p* < 0.05 and ^##^ *p* < 0.01 compared with the mock cells; data are expressed as the mean ± SD, *n* = 6.

After 48 h of transfection, cell cycle distribution was analyzed using flow cytometer. As Figure 4 shows, compared with the control and mock groups, cells of B7-H4 group were arrested in G0/G1 phase.

### 3.5. B7-H4 siRNA Inhibited Invasion and Migration of LOVO Cell Line

**Figure 5:**
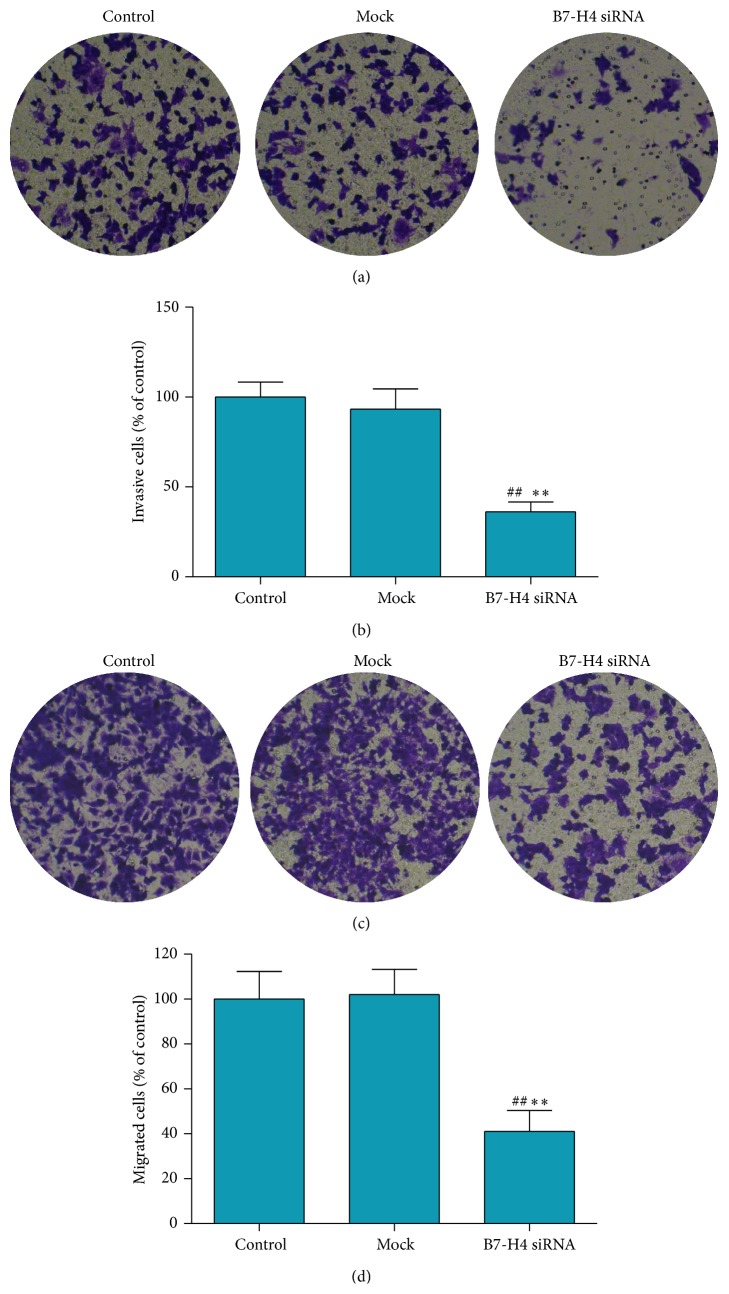
Effect of B7-H4 siRNA on invasion and migration of LOVO cells. (a and b) After B7-H4 siRNA transfection for 48 h, invasive ability of LOVO cells was identified by Transwell assay. (c and d) After B7-H4 siRNA transfection for 48 h, cell migration was identified as previously described. ^*∗∗*^ *p* < 0.01 compared with the control cells; ^##^ *p* < 0.01 compared with the mock cells; data are expressed as the mean ± SD, *n* = 6.

For pervasion to distant organs, the invasion of tumor cells occurs via cell-secreted proteolytic degradation of the cellular basement membrane, which is the leading cause of cancer death [11]. The Transwell Invasion test results are presented in Figures 5(a) and 5(b). Treatment with B7-H4 siRNA for 24 h significantly suppressed the invasion ability of cells when compared with the control group of untreated cells and mock-vehicle group (36.24 ± 5.37% versus 100 ± 8.36% in the control group; 36.24 ± 5.37% versus 93.31 ± 11.24% in the mock group; all *p* < 0.01).

The effects of B7-H4 siRNA on chemotactic motility in cells were evaluated as previously described. As displayed in Figures 5(c) and 5(d), migrated cells decreased dramatically after B7-H4 siRNA transfection treatment for 24 h (41 ± 9.32% versus 100 ± 12.37% in the control group; 41 ± 9.32% versus 102 ± 11.24% in the mock group; all *p* < 0.01). Typical images of B7-H4 siRNA inhibiting cell migration are presented in Figure 5(c).

### 3.6. Identification of Genes and Signaling Associated Biological Pathways and Processes by Gene Set Enrichment Analysis (GSEA)

**Figure 6:**
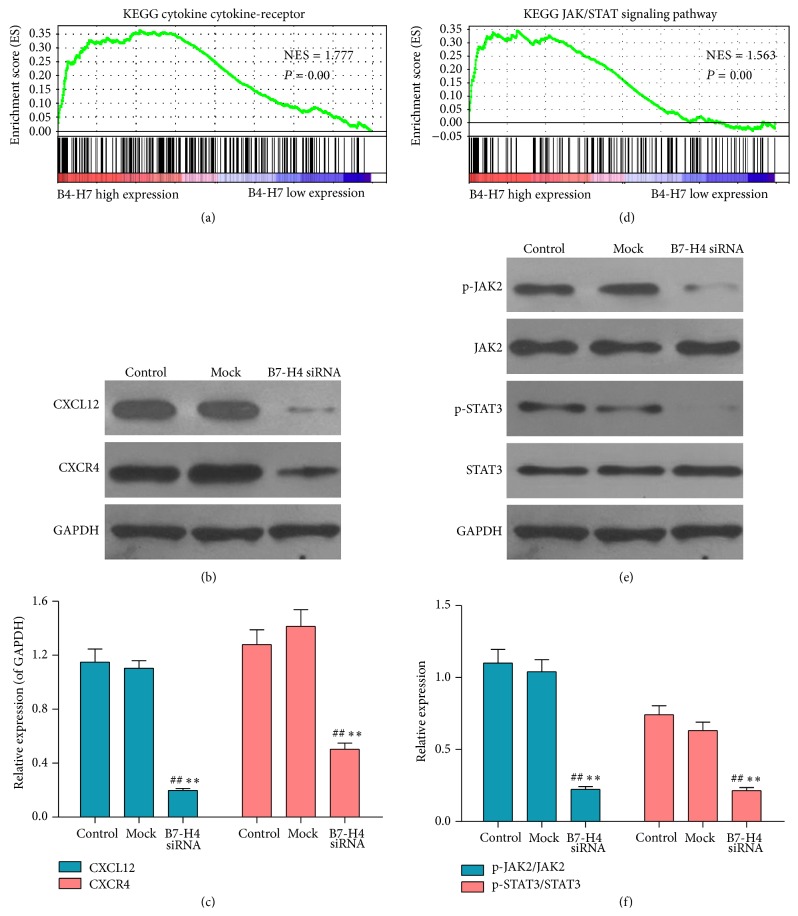
Effect of B7-H4 siRNA on the protein expressions of CXCL12, CXCR4, p-JAK, JAK, p-STAT3, and STAT3 in LOVO cells. (a and d) Identification of CXCL12/CXCR4 and JAK/STAT signalling as regulatory targets of B7-H4. Gene set enrichment analysis (GSEA) identified significant association between B7-H4 and CXCL12/CXCR4 and JAK/STAT signaling pathway in both the TCGA colorectal cancer dataset and the multitumor dataset. (b and c) After 48 h of B7-H4 siRNA treatment, the protein expressions of CXCL12 and CXCR4 in cells were analyzed by western blot. GAPDH was also detected as the control of sample loading. (e and f) Western blot was performed to identify the protein levels of p-JAK2, JAK, p-STAT3, and STAT3, and GAPDH was also detected as the control of sample loading. ^*∗∗*^ *p* < 0.01 compared with the control cells; ^##^ *p* < 0.01 compared with the mock cells; data are expressed as the mean ± SD, *n* = 6.

To probe the B7-H4-associated pathways on an unbiased basis, we performed GSEA using data from the TCGA. GSEA is designed to detect coordinated differences in expression of predefined sets of functionally related genes. Among all the 188 predefined “KEGG pathways” gene sets, the CXCL12/CXCR4 pathway and JAK/STAT pathway were identified with the significant association with B7-H4 expression in the TCGA data (Figures 6(a) and 6(d)).

### 3.7. B7-H4 siRNA Regulated the mRNA Expression of CXCL12 and CXCR4 in LOVO Cells

The effects of B7-H4 siRNA on the mRNA expression of CXCL12 and CXCR4, which are closely related to metastasis of tumor cells, were also investigated and the results are presented in Table 2. As a result, B7-H4 siRNA treatment significantly decreased the expression of CXCL12 and CXCR4 in comparison with the control group and mock group.

### 3.8. B7-H4 siRNA Adjusted the Protein Expression of CXCL12, CXCR4, p-JAK2, and p-STAT3

After 48 h of B7-H4 siRNA treatment, the protein expressions of CXCL12 and CXCR4 in cells were analyzed by western blot. As revealed in Figures 6(b) and 6(c), B7-H4 siRNA significantly decreased the expression levels of CXCL12 (0.1984 ± 0.0146 versus 1.1492 ± 0.0965 in the control group; 0.1984 ± 0.0146 versus 1.103 ± 0.0569 in the mock group; all *p* < 0.01) and CXCR4 (0.5032 ± 0.0459 versus 1.2789 ± 0.1102 in the control group; 0.5032 ± 0.0459 versus 1.4143 ± 0.1236 in mock group; all *p* < 0.01).

After 6 h of B7-H4 siRNA treatment, the phosphorylation protein levels of JAK2 and STAT3 in cells were analyzed by western blot. As shown in Figures 6(e) and 6(f), B7-H4 siRNA significantly decreased the relative expression levels of p-JAK 2/JAK2 (0.2234 ± 0.0197 versus 1.1005 ± 0.0956 in the control group; 0.2234 ± 0.0197 versus 1.04 ± 0.0846 in the mock group; all *p* < 0.01) and p-STAT3/STAT3 (0.2144 ± 0.0214 versus 0.7423 ± 0.0623 in the control group; 0.2144 ± 0.0214 versus 0.6316 ± 0.0594 in mock group; all *p* < 0.01).

### 3.9. B7-H4 siRNA Suppressed Growth of Colorectal Carcinoma In Vivo

**Figure 7:**
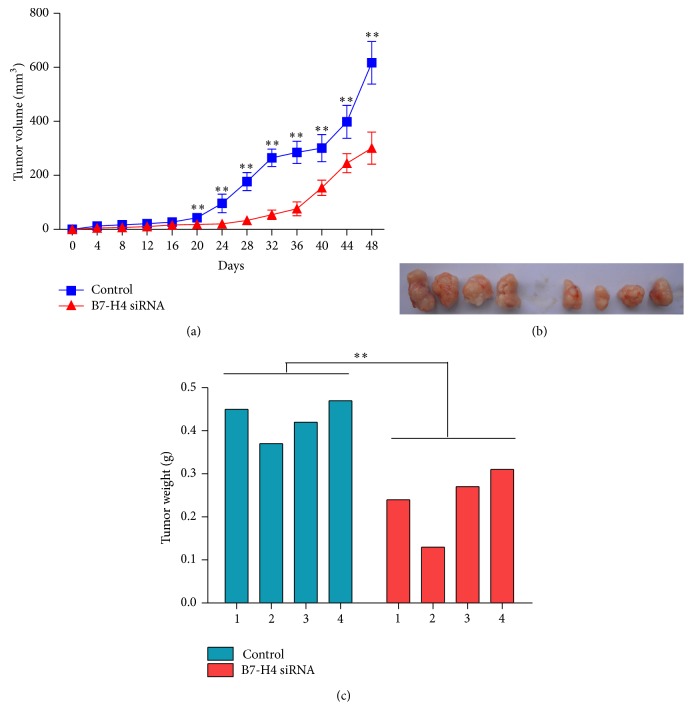
Effect of B7-H4 siRNA on tumor growth in vivo. LOVO cells transfected with B7-H4 siRNA were subcutaneously injected in athymic nude mice. (a) Tumor diameter was evaluated for 48 days. (b) At day 48, mice were sacrificed and tumors were weighted. Tumor growth was significantly reduced in B7-H4 siRNA tumors. ^*∗∗*^ *p* < 0.01 compared with the control cells; data are expressed as the mean ± SD, *n* = 4.

Next, we determined whether silence of B7-H4 in colorectal cancer cells could reduce tumor growth in vivo. LOVO cells transfected with control and B7-H4 siRNA were subcutaneously injected in athymic nude mice, respectively. Tumor volumes were measured for 48 days. As shown in Figure 7(a), B7-H4 silenced tumors grew slower in mice, whereas control tumors grow faster in mice. After 48 days, the volume and weight of B7-H4 siRNA treated tumor were smaller and lighter than those of control (Figure 7(b), *p* < 0.01). These data suggested that inhibition of B7-H4 siRNA in colon cancer cells suppressed tumor growth in nude mice.

## 4. Discussion

B7-H4 is associated with the generation and progression of tumor; it may be a new target in the diagnosis and treatment of tumor. B7-H4 expression has been found in various kinds of tumors including colon, lung, pancreas, brain, stomach, breast, ovary, esophagus, prostate, liver, and kidney [5–8, 12]. Qian et al. demonstrated that B7-H4 siRNA inhibited cell proliferation, colony formation, and migration of pancreatic cancer cells [7]. Shi et al. found that serum B7-H4 expression is a significant prognostic indicator for patients with gastric cancer. Cheng et al. demonstrated that B7-H4 directly promoted malignant transformation of ovarian cancer cell line [13]. In the present study, we found that B7-H4 is highly expressed in colorectal tumor tissues.

Among several cell lines, B7-H4 was found to be notably expressed in LOVO cells. Therefore, LOVO cell line was determined for further investigations. B7-H4 siRNA effectively inhibited proliferation, invasion, and migration of LOVO cells. In order to elucidate the possible mechanism involved, we performed GSEA to identify associated biological processes and signaling pathways using the mRNA signature based risk score for classification. The risk score was accompanied with the downregulation of several cancer-related networks, namely, CXCL12/CXCR4 pathway and JAK/STAT. These indicated that CXCL12/CXCR signaling and JAK/STAT signaling play crucial role in the process of antiproliferation, anti-invasion, and antimigration triggered by B7-H4. Further in vivo tumor formation study in nude mice indicated that the inhibition of B7-H4 siRNA in colorectal cancer cells delayed the progress of tumor formation. These results suggested that B7-H4 might be a potential therapy target for colorectal cancer.

The chemokine CXCL12 is an extracellular chemokine which binds to its cell surface receptor CXCR4. Various CXCR4/CXCL12 axis-related intracellular signal transduction cascades and effectors, important regulators for survival, proliferation, and death have been determined, such as MAPK [14], JAK/STAT, PI-3K/Akt, and Wnt [15–17]. The axis of CXCL12/CXCR4 has been considered to play an important role for cancer cell migration [15]. Zhong et al. reported that CXCL12/CXCR4 axis played an important role in the progression and organ-specific metastasis of pancreatic adenocarcinoma [18]. Wu et al. demonstrated that tumor cells from pancreatic cancer express high level of CXCR4 [19]. CXCL12 is the ligand for CXCR4, which is extensively secreted by neighboring stromal cells and other distant organs. CXCL12 primarily binds to CXCR4 and induces intracellular signaling through several divergent pathways, which are involved in progression and metastasis of cancer. Xie et al. investigated that the specific downregulation of CXCR4 inhibited cell growth, invasiveness, and migration of NSCLC. The accumulating reports have shown that CXCL12 and CXCR4 are overexpressed both in mRNA and protein levels during initiation and progression of cancers. In our study, B7-H4 siRNA significantly suppressed the mRNA and protein expressions of CXCL12 and CXCR4, which could interpret the effects of antiproliferation, anti-invasion, and antimigration of B7-H4 in LOVO cells.

In addition, phosphorylation of JAK/STAT signaling was found negatively adjusted by B7-H4 siRNA. STAT3, an important transcription factor of JAK/STAT signal transduction pathway, can promote the rapid induction of genes by directly transducing signals from the receptor into the nucleus and play a pivotal role in mediating the biological response for these ligands. Activation of the signal transcription-3 (STAT3) signal transduction pathway largely contributes to inflammation and carcinogenesis. Increasing evidence displayed that constitutive activation of STAT3 gave rise to neoplasm invasion and metastasis [20, 21]. In the present study, the phosphorylation of JAK2 and STAT3 was dramatically inhibited, which was also the mechanism of antiproliferation, anti-invasion, and antimigration of B7-H4 siRNA.

## 5. Conclusions

In conclusion, siRNA targeting of B7-H4 effectively suppressed the proliferation, invasion, and migration of LOVO cells by inhibiting the effect of CXCL12/CXCR4 and the phosphorylation of JAK2/STAT3 on increasing the metastatic potential of colorectal carcinoma. This work can supply important evidence for the prevention and treatment of colorectal cancer.

## Supplementary Material

Real-time PCR was performed to detect the expression levels of B7-H4 in colon cancer tissues and ovarian cancer tissues. All the tissue samples were collected from Shanghai Tongren Hospital.

## Figures and Tables

**Table 1:** Primers used in FQ-RT-PCR analysis.

Gene	Primer sequence	Species	Amplicon size (bp)
B7-H4	Forward: 5′-AGGGAGTGGAGGAGGATACAG-3′ Reverse: 5′-GCAGCAGCCAAAGAGACAG-3′	Human	137
CXCL12	Forward: 5′-CCAAGTAAOCAGGGCAGGAG-3′ Reverse: 5′-AGGTGAGGGGAAAACAAACAA-3′	Human	152
CXCR4	Forward: 5′-GCCAACGTCAGTGAGGCAGA-3′ Reverse: 5′-AACCATGATGTGCTGAAACTGGAA-3′	Human	249
GAPDH	Forward: 5′-CACCCACTCCTCCACCTTTG-3′ Reverse: 5′-CCACCACCCTGTTGCTGTAG-3′	Human	110

**Table 2:** Effects of B7-H4 siRNA on the mRNA expression of CXCL12 and CXCR4.

Group	CXCL12 (%)	CXCR4 (%)
Control	4.90 ± 0.0038	8.93 ± 0.0163
Mock	4.66 ± 0.0084	7.44 ± 0.0043
B7-H4 siRNA	1.54 ± 0.0042^∗∗##^	2.48 ± 0.0035^∗∗##^

After LOVO cells were treated with B7-H4 siRNA transfection for 6 h; the RNA expression of CXCL12 and CXCR4 was detected by RT-PCR. ^∗∗^ *p* < 0.01 compared with the control group; ^##^ *p* < 0.01 compared with the mock group; data are expressed as the mean ± SD, *n* = 6.
