# Supplementary material for: Role of B7-H4 siRNA in Proliferation, Migration, and Invasion of LOVO Colorectal Carcinoma Cell Line
Source: Biomed Res Int. 2015 May 21;2015:326981. doi: 10.1155/2015/326981 (PMC4454715; doi:10.1155/2015/326981)
Supplement: Supplementary file 1 — Real-time PCR was performed to detect the expression levels of B7-H4 in colon cancer tissues and ovarian cancer tissues. All the tissue samples were collected from Shanghai Tongren Hospital. [file 326981.f1.docx]

Figure S1. **B7-H4 expression in colon cancer and ovarian normal tissues.**

10 pairs of colon cancer tissues and their adjacent normal tissues, and 10 pairs of ovarian cancer tissues and their adjacent normal tissues were collected. mRNA expression of B7-H4 mRNA expression was identified by RT-PCR.
